# Entropy Generation Analysis and Performance Comparison of a Solid Oxide Fuel Cell with an Embedded Porous Pipe Inside of a Mono-Block-Layer-Build Geometry and a Planar Geometry with Trapezoidal Baffles

**DOI:** 10.3390/e27070659

**Published:** 2025-06-20

**Authors:** J. J. Ramírez-Minguela, J. M. Mendoza-Miranda, V. Pérez-García, J. L. Rodríguez-Muñoz, Z. Gamiño-Arroyo, J. A. Alfaro-Ayala, S. Alonso-Romero, T. Pérez-Segura

**Affiliations:** 1 Department of Chemical Engineering, Division of Natural and Exact Sciences, University of Guanajuato, Col. Noria Alta s/n, Guanajuato C.P. 36050, Guanajuato, Mexico; 2 Unidad Profesional Interdisciplinaria de Ingeniería Campus Guanajuato, Instituto Politécnico Nacional, Av. Mineral de Valenciana 200 Fracc, Industrial Puerto Interior, Silao de la Victoria C.P. 36275, Guanajuato, Mexico; 3 Department of Mechanical Engineering, Engineering Division Campus Irapuato-Salamanca, University of Guanajuato, Salamanca-Valle de Santiago km 3.5+1.8, Salamanca C.P. 36885, Guanajuato, Mexico; 4 Ingeniería Mecánica, Escuela Superior de Ciudad Sahagún, Universidad Autónoma del Estado de Hidalgo, Carretera Cd. Sahagún-Otumba s/n, Zona Industrial, Ciudad Sahagún C.P. 43970, Hidalgo, Mexico; 5 CIATEC, A.C., Centro de Innovación Aplicada en Tecnologías Competitivas, Omega 201, Col. IndustrialDelta, León C.P. 37545, Guanajuato, Mexico

**Keywords:** MOLB-PPA, porous pipe, P-TBFA, trapezoidal baffles, CFD, entropy generation analysis

## Abstract

An analysis of entropy generation and a performance comparison are carried out for a solid oxide fuel cell with an embedded porous pipe in the air supply channel of a mono-block-layer-build geometry (MOLB-PPA SOFC) and a planar geometry with trapezoidal baffles inside the fuel and air channels (P-TBFA SOFC). The results for power density at different current densities are discussed. Also, a comparison of the field of species concentration, temperature, and current density on the electrode–electrolyte interface is analyzed at a defined power density. Finally, a comparison of maps of the local entropy generation rate and the global entropy generation due to heat transfer, fluid flow, mass transfer, activation loss, and ohmic loss are studied. The results show that the MOLB-PPA SOFC reaches a 7.5% higher power density than the P-TBFA SOFC. Furthermore, the P-TBFA SOFC has a more homogeneous temperature distribution than the MOLB-type SOFC. The entropy generation analysis indicates that the MOLB-PPA SOFC exhibits lower global entropy generation due to heat transfer compared to the P-TBFA SOFC. The entropy generation due to ohmic losses is predominant for both geometries. Finally, the total irreversibilities are 24.75% higher in the P-TBFA SOFC than in the MOLB-PPA SOFC.

## 1. Introduction

Fossil fuels are still the main resource to produce energy, and this provokes an increment in greenhouse gases and other harmful consequences. To reduce these phenomena, several research are focused on improving more environmentally friendly technologies. In this sense, the use of fuel cells can be useful to reduce this effect due to their versatility. Among the different fuel cell technologies, solid oxide fuel cells are distinguished for their capability to be used in residential applications or power plans due to their higher energy conversion efficiencies, lower production costs, and high operating temperatures, 873–1273 K [1,2,3,4], that help to be used in cogeneration or trigeneration systems [5,6]. Singh and Singh [7] studied a cogeneration system based on combining SOFC with a humid air turbine and supercritical carbon dioxide cycle for power and heating. They performed a parametric analysis considering a thermodynamic model. Their results showed the advantages of using the cogeneration system. Wang and Hou [8] compared tri-reforming in SOFC systems. Four system configurations and two reformer types were studied. The results obtained show better performance, higher electric efficiency of the system, and a decrease in the max temperature gradient in the SOFC. Tian et al. [9] analyzed a cogeneration system that uses the heat of the SOFC in a bi-evaporator organic flash cycle. Thermodynamic, environmental, and economic aspects of various organic fluids were evaluated. The results showed an improvement in the cycle using the SOFC.

Furthermore, there are some challenges that need to be solved before reaching mass marketing. In this regard, the high temperature gradients reached inside the SOFC components are relevant and could provoke failures in the materials of the components due to thermal stresses [10,11,12,13,14,15]. Yang et al. [16] compared the planar geometry and MOLB SOFC SOFCs. The heat, the mass transfer, and the working conditions were analyzed. High temperature gradients were obtained in both geometries. Tseronis et al. [17] developed a multidimensional model of non-isothermal planar SOFC. Species profiles, temperature profiles, current density distribution, and polarization curves were predicted using the model. High temperature gradients were found within the SOFC. Ho [18] analyzed the transient characteristics of an anode-supported SOFC by means of a three-dimensional model. A repeating unit of a planar anode-supported SOFC with co-flow configuration was investigated. Its results showed high temperature gradients along the cell at different time-steps in the transient profiles of the temperature. Choudhary and Sanjay [19] developed a three-dimensional anode-supported internal reforming planar SOFC model. They analyzed the fuel and air composition, temperature, voltage, and current density related to the electrochemical model of the fuel cell. Also, they studied the effect of co-flow and counter-flow configurations on cell performance. Their results showed high temperature gradients for the co-flow and the counter-flow configurations. Xu et al. [20] developed a model to study thermal stress in SOFCs using the finite element method. They analyzed the thermal stress in different operating situations in the SOFC. Their results show high temperature gradients and discuss in detail the effects of thermal stress on the SOFC components.

On the other hand, as it is well known, a reduction in the temperature gradient in solid oxide fuel cells could improve their stability. Therefore, some works focused on reducing the high temperature gradients inside the SOFC, such as Kim et al. [21], who studied the effect of two different design factors on the heat transfer mechanism of a 1 kW planar SOFC stack by means of three-dimensional thermo-fluid simulations. They discussed the improvement in terms of average temperature, temperature gradients, and thermal load that was achieved in their proposed interconnect design. Sezer et al. [22] developed a three-dimensional transient model to predict the performance degradation of planar SOFC anodes due to the transport and chemical deposition of the fuel contaminant phosphine exposed to fuel contaminants commonly present in coal syngas. Their results show the temperature distribution in the fuel channel from inlet to outlet for different phosphine exposure times. They found that the temperature gradient increases significantly with time. Hao et al. [23] analyzed the temperature gradient reduction of a 2D-axisymmetric model of a micro-tubular SOFC. They include in the SOFC tubular separators and flanged separators. They demonstrated that both separators reduce the temperature gradient of the cell. Ramírez-Minguela et al. [24] made a detailed comparison of the performance of a simple mono-block-layer-build-type SOFC geometry and a mono-block-layer-build-type SOFC with an embedded porous pipe in the air supply channel considering different porosities in the pipe. Their results showed a more homogeneous temperature distribution for the cases that consider the porous pipe. Ramírez-Minguela et al. [25] studied numerically the effect of including trapezoidal baffles inside fuel and air channels of a simple planar geometry. Their results showed a significant reduction in the temperature gradients inside the SOFC for the case with the inclusion of baffles inside the air channel and for the case with the baffles inside both channels. Shi et al. [26] proposed a new series connection structure for a tubular solid oxide fuel cell. They studied the effects of the series structure on the distribution of species, average temperature, and electrical properties of the cell. Their results showed an increase in the average current density considering their proposal compared to traditional tubular SOFC. Also, the maximum temperature was significantly reduced after they added a cooling channel.

Moreover, studies on irreversibilities due to the multiple physical phenomena that occur inside the SOFC are scarce [27]. Choudhary and Sanjay [28] optimized the performance of a hybrid system that consists of the integration of a fuel cell with a conventional gas turbine. They analyzed the influence of operating temperature recirculation on cell performance, as well as the influence of turbine inlet temperature on blade coolant requirement, the influence of compression ratio, and turbine inlet temperature on the efficiency of the hybrid system. They developed a constraint-based algorithm to optimize the hybrid system by entropy generation minimization. It is worth mentioning that the entropy analysis considers a black box model; the local entropy generation rate of each device that makes up the system was not calculated. Ramírez-Minguela et al. [29] analyzed the entropy generation in a MOLB-type SOFC using hydrogen with different operating conditions and different electrolyte thicknesses. The investigation was based on a three-dimensional CFD model. Their results show high temperature gradients along the cell and strong variations in the local and global entropy generation values. Ramírez-Minguela et al. [30] calculated the local and global entropy generation rate in a 3D model that considers biogas as a fuel in an SOFC from different sources. From their results, they found that the main irreversibility is due to the activation losses and reaches up to 40% of the global entropy generation. Gedik et al. [31] studied a 1D model based on the non-equilibrium thermodynamics for a single solid oxide fuel cell, considering extended mass transport mechanisms based on the Dusty-Gas model and thermal diffusion (Sore effect) in the gas diffusion layers. They analyzed the local entropy production rate in the anode, the cathode, and the electrolyte. They found that the main irreversibilities are in the electrolyte, 64.44% of the total losses in the cell. Lai et al. [32] analyzed various system designs and parameters to enhance the efficiency of SOFC systems. They calculated the entropy, black box model, of the effects of design configuration under different anode off-gas recirculation and fuel utilization conditions to improve the efficiency of the SOFC system. Their results show that the temperature difference is the key factor influencing the entropy of heat components in the SOFC system. In this sense, as was previously described, due to the operational high temperature of the SOFCs, the entropy generation analysis in SOFC could help to improve heating and cooling strategies.

Therefore, in this work, a detailed comparison of the entropy generation and performance in terms of power density, fluid flow, species concentration, temperature and electric fields inside the cell of a modified mono-block-layer-build with an embedded porous pipe in the air supply channel (MOLB-PPA SOFC), and a modified planar geometry with trapezoidal baffles inside fuel and air channels (P-TBFA SOFC), in a solid oxide fuel cell (SOFC) is carried out by means of a three-dimensional numerical model using Computational Fluid Dynamics (CFD). The numerical model takes into account the physical phenomena inside the fuel cell, such as heat transfer, mass transfer, species transport, and electrochemical reactions. For the MOLB-PPA SOFC, a constant porosity along the porous pipe is considered. For the P-TBFA SOFC, the height of the baffles inside the channels of the solid oxide fuel cell increases along the direction of the fluid flow.

## 2. Model Geometry

**Figure 1:**
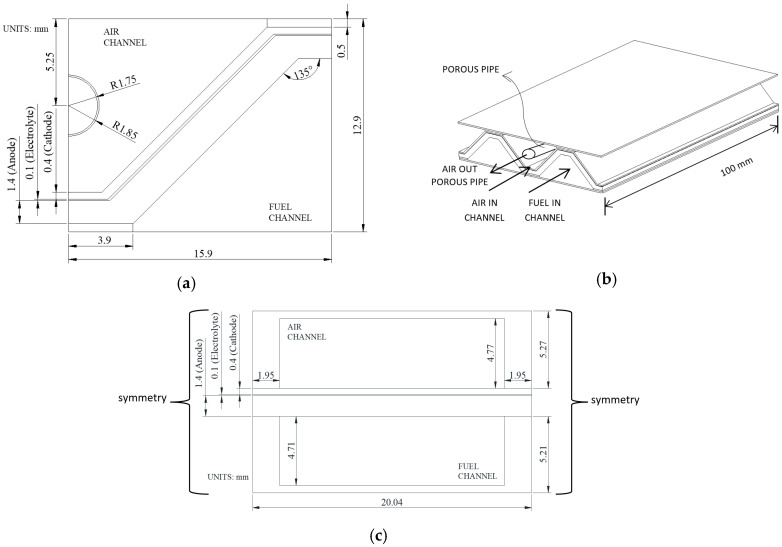

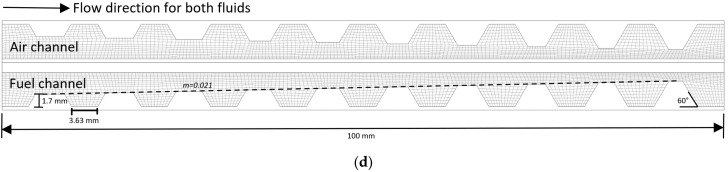
Geometry dimensions: (**a**) MOLB-PPA SOFC transversal section, (**b**) MOLB-PPA SOFC length, (**c**) P-TBFA SOFC transversal section, (**d**) configurations along the P-TBFA SOFC.

The geometries and their dimensions for the three-dimensional numerical model are shown in Figure 1. The domains of the geometries consider the electrodes, the electrolyte, the fuel and air channels, and the current collectors. The porous pipe of the MOLB-PPA SOFC is fed with a flow of air in a counter-flow arrangement with respect to the flow of the air channel (Figure 1b). The height of the trapezoidal baffles inside the channels of the P-TBFA SOFC increases along the direction of the fluid flow (Figure 1d). A parallel flow arrangement for the fuel and the air channels is considered in both geometries (Figure 1). As illustrated, both geometries share the same anode, electrolyte, and cathode thicknesses and lengths. The active area, defined by the anode–electrolyte and cathode–electrolyte interfaces, is also identical in both cases. Additionally, the cross-sectional dimensions of the air and fuel channels are kept the same to ensure a fair and valid comparison between the two designs.

According to Figure 1, the cases analyzed are as follows:

- Case 1: MOLB-PPA SOFC with an embedded porous pipe with a constant porosity (Figure 1a,b);
- Case 2: P-TBFA SOFC with trapezoidal baffles along the direction of the fluid flow in the fuel channel and the air channel (Figure 1c,d).

## 3. Numerical Model

### 3.1. Continuity, Momentum, and Energy Equations

The differential partial equations that consider the velocity of the fluid, species, and temperature of the SOFC are the following [33]:

(1) $$\nabla \cdot \rho \vec{v}=0$$

(2) $$\nabla \cdot \left(\rho \vec{v}\vec{v}\right)=-\nabla \cdot p+\nabla \cdot \left(\overset{̿}{\tau }\right)+\rho \vec{g}+S_{m}$$

(3) $$S_{m}=-\left(\frac{\mu }{K}v_{i}\right)$$

(4) $$\nabla \cdot \left(\rho \vec{v}\left(h+\frac{v^{2}}{2}\right)\right)=\nabla \cdot \left(k_{eff}\nabla T-\sum_{\alpha }h_{\alpha }{\vec{J}}_{\alpha }\right)+S_{e}$$

(5) $$k_{eff}=\varepsilon \cdot k_{g}+\left(1-\varepsilon \right)\cdot k_{s}$$

(6) $$\nabla \cdot \left(\rho \omega_{i}\vec{v}\right)=-\nabla \cdot {\vec{J}}_{i}+S_{i}$$

(7) $${\vec{J}}_{i}=-\rho D_{i,eff}\nabla \omega_{i}$$

(8) $$D_{i,eff}=\frac{\varepsilon }{ϯ}D_{ij}$$

The conservation of mass (Equation (1)) is necessary to solve the fluid flow part, considering the flow as incompressible. The conservation of momentum (Equation (2)) takes into account the use of laminar flow through the porous medium as the anode and the cathode of the SOFC by means of the source term described in Equation (3). To solve the local temperature inside the SOFC, the energy equation (Equation (4)) needs to be considered. For simulations, the porous medium and fluid flow are assumed to be in thermal equilibrium; therefore, the conduction flux in the porous medium uses an effective conductivity, $k_{eff}$ (Equation (5)). To solve and predict the local mass fraction of each species, i, in the domains of the model, it is necessary to consider the species transport equation (Equation (6)), where $S_{i}$ is the rate of creation or consumption of the species due to the electrochemical reactions defined by means of user-defined functions. ${\vec{J}}_{i}$ is the diffusion flux defined by Equation (7). The effective binary diffusivity, $D_{i,eff},$ is defined in Equation (8), where the binary diffusivity, $D_{ij}$, is obtained through the Fuller–Schettler–Gidding correlation. In the fuel and air channels, $D_{i,eff}$ coincides with $D_{ij}$, while in the porous media, the effective binary diffusivity is computed as Equation (8).

### 3.2. Electrochemical Model

Moreover, to solve the electrochemical reactions, species consumption, heat release due to the electrochemical reactions, and the main losses in an SOFC, it is necessary to solve the Equations (9)–(22) [34,35]. The details of the variables can be found in the nomenclature section.

(9) $$\frac{1}{2}O_{2}+2e^{-}\to O^{2-}$$

(10) $$H_{2}+O^{2-}\to H_{2}O+2e^{-}$$

(11) $$H_{2}+\frac{1}{2}O_{2}\to H_{2}O$$

(12) $$S_{i}=\left\{\begin{array}{cc} \frac{j}{n_{e}F}M_{i} & anode\;and\;cathode\\ 0 & elsewhere \end{array}\right.$$

(13) $$j=j_{0}\cdot \left\{exp\left[\frac{\beta \cdot n_{e}\cdot F}{R\cdot T}n_{act}\right]-exp\left[-\frac{\left(1-\beta \right)\cdot n_{e}\cdot F}{R\cdot T}n_{act}\right]\right\}$$

(14) $$S_{e,act}=j\cdot n_{act}$$

(15) $$S_{e,elec}=\frac{j}{n_{e}F}T∆S$$

(16) $$S_{e,ohm}=\frac{j^{2}}{\sigma }$$

(17) $$S_{e}=S_{e,act}+S_{e,elec}+S_{e,ohm}$$

(18) $$\nabla \cdot \left(\sigma \nabla \emptyset \right)=\left\{\begin{array}{cc} -j & TPB\\ 0 & elsewhere \end{array}\right.$$

(19) $$V_{rev}=V^{0}+\frac{RT}{2F}ln\left(\frac{p_{H_{2}}\sqrt{p_{O_{2}}}}{p_{H_{2}O}}\right)$$

(20) $$V^{0}=1.317-2.769\cdot 10^{-4}\cdot T$$

(21) $$V=V_{rev}-n_{act}-n_{ohm}-n_{conc}$$

(22) $$n_{ohm}=jR_{ohm}$$

Hydrogen is supplied at the fuel channel inlet, and air is supplied at the air channel inlet. Therefore, the electrochemical reactions that take place in the anode and cathode sides are defined by Equations (9)–(11). The consumption and the production of species that consider Equation (6) are obtained from the species volumetric source terms (Equation (12)) due to the electrochemical reactions, where on the cathode side, the oxygen is consumed, and on the anode side, the hydrogen and the steam are consumed and produced, respectively. $n_{e}$ is the number of electrons per mole of reactants and products. $M_{i}$ is the molecular mass of hydrogen, oxygen, and steam. F is the Faraday constant. j is the current density defined by the Buttler–Volmer equation (Equation (13)), where $j_{0}$ is the exchange current density, β is the transfer coefficient. Moreover, the driving force for kinetics is the local surface overpotential, $n_{act}$, also known as the activation loss, and it is considered in the electrochemical reactions in the anode and the cathode sides. Due to the irreversibilities of the process, all the chemical energy released in the electrochemical reactions cannot be converted to electrical work; therefore, the activation loss provokes an increase in the temperature of the anode–electrolyte–cathode of the SOFC, and it is defined as an energy source term (Equation (14)) on the energy equation (Equation (4)). Moreover, the heat released due to the electrochemical reaction is considered in Equation (15), and the heat released due to the ohmic resistivity of the conducting media as the current density is generated is considered in Equation (16), where σ is the electrical conductivity of the materials of the SOFC [36]. Therefore, the total energy source term is defined in Equation (17). Moreover, Equation (18) is considered to obtain the electric potential of the cell, where ∅ is the electric potential and TPB is the three-phase boundary. The reversible potential, $V_{rev}$, is defined by Equation (19), where $V^{0}$ is the standard electric potential defined by Equation (20). The operating voltage of the cell, V, that considers the main losses of the cell is defined in Equation (21), where $n_{ohm}$ is the voltage loss due to the internal resistance of the materials of the SOFC, anode, electrolyte, cathode, and connections, and it is defined by Equation (22), where $R_{ohm}$ is the total resistance.

Lastly, the model parameters used to solve the numerical model are reported by Yang et al. [16] (Table 1).

### 3.3. Entropy Generation Formulations

For the entropy generation analysis, for both geometries, it is necessary to solve the following equations [29]:

(23) $$s_{h}=\frac{k{\left(\nabla T\right)}^{2}}{T^{2}}$$

(24) $$s_{\mu }=\frac{∆:\tau }{T}$$

(25) $$s_{m}=\sum_{i}\left(\rho R_{i}D_{i}\right)\left(\nabla \omega_{i}\cdot \frac{\nabla X_{i}}{X_{i}}\right)$$

(26) $$s_{act}=\frac{j\cdot n_{act}}{T}$$

(27) $$s_{ohm}=\frac{1}{T}\left(\frac{j^{2}}{\sigma }\right)$$

(28) $$S_{i}=\int s_{i}dV$$

(29) $$S_{i,act}=\int s_{act}dA$$

Equation (23) considers the local entropy generation rate due to heat transfer. Equation (24) considers the local entropy generation rate due to fluid flow and viscous stress, where ∆ and τ are the strain and stress tensors, respectively. Moreover, the local entropy generation rate due to mass transfer is considered in Equation (25), where $R_{i}$, $D_{i}$, $\omega_{i},$ and $X_{i}$ are the universal gas constant, the mass diffusive coefficient, the mass fraction, and the molar fraction of the species i, respectively. The local entropy generation rate due to activation loss is calculated as shown in Equation (26), and it is considered a surface term on the anode–electrolyte and cathode–electrolyte interfaces. Furthermore, the local entropy generation rate due to ohmic loss is calculated as shown in Equation (27). Equation (28) is used to obtain the global entropy generation due to heat transfer, fluid flow, mass transfer, and ohmic loss. Finally, Equation (29) is used to obtain the global entropy generation due to activation loss.

### 3.4. Boundary Conditions and Numerical Implementation

For the MOLB-PPA SOFC, a velocity magnitude of 3.0 m/s and a temperature of 1073 K are imposed at the inlet of the air channel and at the inlet of the embedded porous pipe. For the P-TBFA SOFC, a velocity magnitude of 0.5 m/s and a temperature of 1073 K are applied at the inlet of the fuel channel. For the air channel inlet for both geometries and the inlet of the embedded porous pipe on the MOLB-PPA SOFC, an O_2_ mass fraction of 0.2329 is considered. For both geometries, a H_2_ mass fraction of 0.4752 is considered in the inlet fuel channel. The limit of the average temperature on the electrode–electrolyte interfaces at which the SOFC can be operated was restricted to 1273 K [1,2,3,4]. Atmospheric pressure is considered at the outlet cross-sections of the geometries (fuel outlet channels of both geometries, air outlet channels of both geometries, and air outlet of the embedded porous pipe of the MOLB-PPA SOFC). For both geometries, SOFC is considered adiabatic, and a symmetry boundary condition is considered on the left and right sides of the SOFC (Figure 1a,c).

The commercial Computational Fluid Dynamics (CFD) software (ANSYS-FLUENT^®^ v.18.1) based on the finite volume method as its discretization technique is considered to solve the equations described in the previous section. For the spatial discretization, the Green–Gauss cell-based is used for the gradient, while for the pressure, the standard approximation, for momentum, energy, electric potential, and the species involved in the model, the first-order upwind scheme is used. The porosity of the anode and cathode are considered homogeneous and isotropic. A thermal equilibrium between solid and gas phases is considered. For running the model, hybrid initialization is used as the initialization method.

The variable output used for mesh independence testing was the average current density at the anode–electrolyte interface. Simulations were performed using different mesh sizes for both geometries. The variation in average current density with increasing mesh element count is presented in Table 2. The results indicate that mesh sizes of approximately 290,000 elements for the MOLB-PPA SOFC and 3,000,000 elements for the P-TBFA SOFC were sufficient to achieve mesh-independent solutions, with variations of less than 1% upon further mesh refinement. Moreover, model validation was performed by applying the previously described equations and considering the parameters and operating conditions reported in the literature [16,37,38,39]. The simulation results showed good agreement in terms of species distribution, temperature profiles, and current density values, exhibiting similar trends and numerical behavior as those reported in the referenced studies. Finally, the entropy generation analysis is carried out as a postprocess of the results obtained for each simulation.

## 4. Results and Discussion

**Figure 2:**
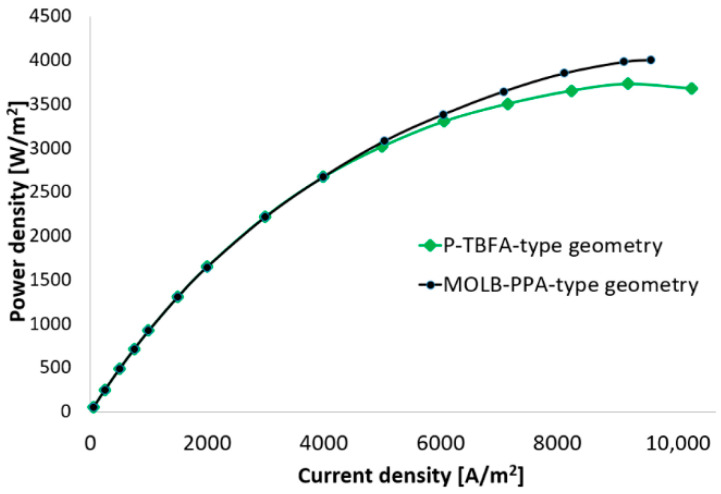
Power density at different current densities.

The performance in terms of power density of the SOFC considering a planar geometry with trapezoidal baffles inside fuel and air channels (P-TBFA SOFC) and the modified mono-block-layer-build with an embedded porous pipe in the air supply channel (MOLB-PPA SOFC) is shown in Figure 2. As can be observed, at lower current densities (<4000 A/m^2^), both geometries have similar performance. Moreover, at current densities higher than 4000 A/m^2^, the MOLB-PPA SOFC has a better performance in terms of power density than the P-TBFA SOFC. In this regard, as the current density increases, the performance of the MOLB-PPA SOFC is more significant, i.e., for the current density of 6000 A/m^2^, the power density for the P-TBFA SOFC is around 3300 W/m^2^, while for the MOLB-PPA SOFC, it is around 3390 W/m^2^, this means that the MOLB-PPA SOFC has an increase of around 3%. Moreover, for a current density of 9200 A/m^2^, the power density for the P-TBFA SOFC is around 3700 W/m^2^ (this is the maximum power density reached for this geometry), while for the MOLB-PPA SOFC, it is around 3980 W/m^2^, this means that the MOLB-PPA SOFC has an increase of around 7%. In this sense, it is worth mentioning that the maximum power density reached for the MOLB-PPA SOFC is around 4010 W/m^2^. This means an increase of around 7.5% with respect to the maximum power density reached for the P-TBFA SOFC.

**Figure 3:**
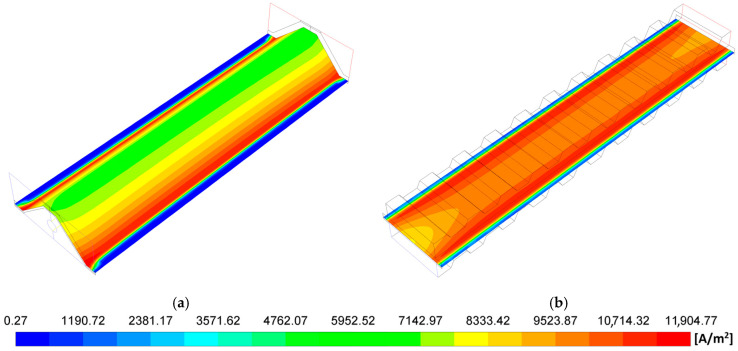
Current density distribution in the electrode–electrolyte interfaces: (**a**) MOLB-PPA SOFC and (**b**) P-TBFA SOFC.

**Figure 4:**
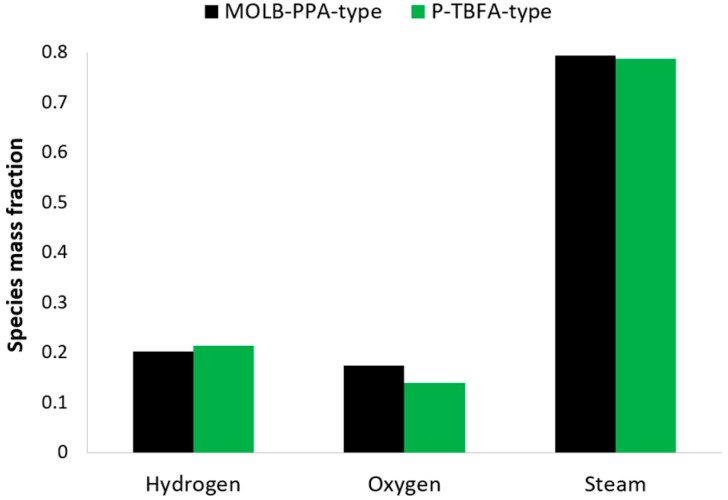
Species mass fraction for both geometries.

**Figure 5:**
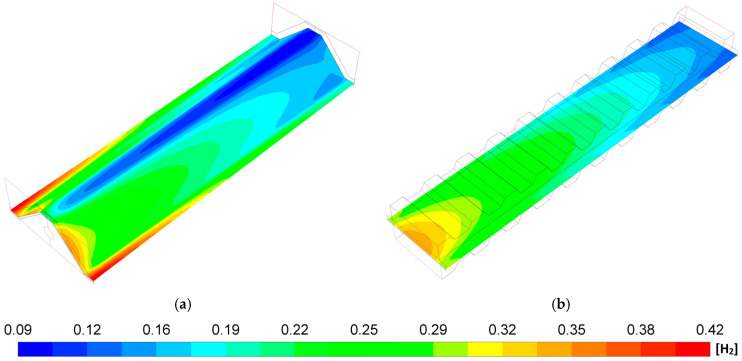
H_2_ mass fraction distributions in the anode–electrolyte interface: (**a**) MOLB-PPA SOFC and (**b**) P-TBFA SOFC.

**Figure 6:**
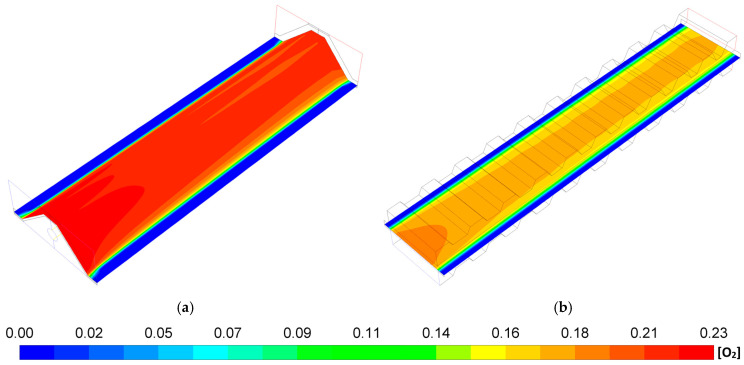
O_2_ mass fraction distributions in the cathode–electrolyte interface: (**a**) MOLB-PPA SOFC and (**b**) P-TBFA SOFC.

**Figure 7:**
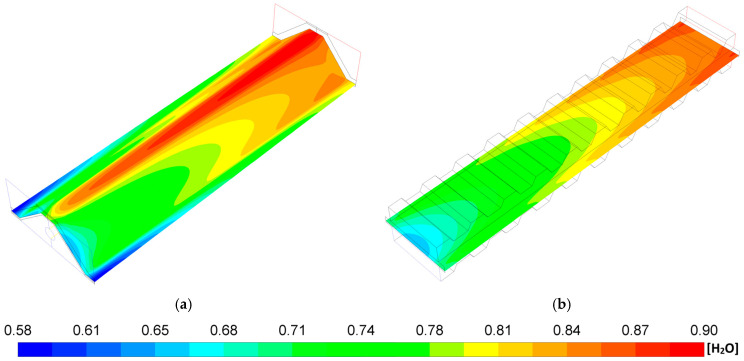
H_2_O mass fraction distributions in the anode–electrolyte interface: (**a**) MOLB-PPA SOFC and (**b**) P-TBFA SOFC.

**Figure 8:**
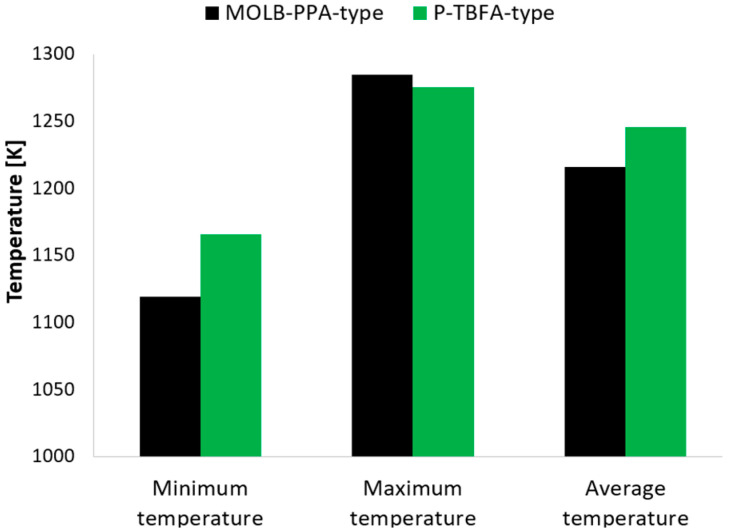
Minimum, maximum, and average temperatures for both geometries.

Figure 3, Figure 4, Figure 5, Figure 6, Figure 7 and Figure 8 show a comprehensive comparison of the MOLB-PPA SOFC and the P-TBFA SOFC in terms of current, species concentration, and temperature. The maximum power density of 3700 W/m^2^ reached for the case of the P-TBFA SOFC is considered for the comparison of both geometries.

The current density distribution for MOLB-PPA SOFC and P-TBFA SOFC is shown in Figure 3. As can be observed on the anode–electrolyte interface, the current density of the P-TBFA SOFC is higher than that in the MOLB-PPA SOFC. Therefore, the average current density necessary for the MOLB-PPA SOFC to reach the power density of 3700 W/m^2^ is lower than the P-TBFA-type, around 18.5%, while the P-TBFA SOFC needs an average value of the current density of 9200 A/m^2^, the MOLB-PPA SOFC needs an average value of 7490 A/m^2^. Moreover, the lower values of the current density distribution for MOLB-PPA SOFC occur on the lower surface where the inclined plane of the anode begins. The lower values for P-TBFA SOFC take place where the anode, electrolyte, cathode, and collectors are connected. These zones with the lower values are related to the H_2_ and O_2_ mass fractions distribution in the anode–electrolyte interface and the cathode–electrolyte interface, respectively (Figure 5 and Figure 6).

The average mass fraction availability of the species of H_2_, O_2,_ and H_2_O on the electrode–electrolyte interfaces, considering the maximum power density in both geometries, is illustrated in Figure 4. The MOLB-PPA SOFC shows a lower H_2_ mass fraction than the P-TBFA SOFC, around 4.6%. This phenomenon is strongly related to the average current density needed to obtain the power density desired. At lower current density, higher H_2_ availability on the anode–electrolyte should be obtained; however, in this case, the MOLB-PPA SOFC has the lowest average current density. In this sense, it can be said that the H_2_ species distribution on the anode–electrolyte interface has a more meaningful effect in this geometry than in the P-TBFA SOFC (Figure 5). Furthermore, the average O_2_ mass fraction for the MOLB-PPA SOFC is around 0.1733, while for the P-TBFA SOFC is around 0.1392. This means that the inclusion of the porous pipe achieves an increase in the O_2_ mass fraction concentration of around 24.5%. This phenomenon is due to the counter-flow arrangement between the porous pipe and the air channel supply. As the O_2_ mass fraction is consumed due to the electrochemical reactions that occur inside the cell to produce the current density of the cell, the O_2_ mass fraction feed in the porous pipe helps replace the consumption of the O_2_ near the outlet of the air channel. Lastly, a similar steam production for the MOLB-PPA SOFC is obtained in comparison to the P-TBFA SOFC, a difference of around 1%. This difference is related to the phenomena described above for the H_2_ mass fraction availability. Finally, the concentration losses depend on the availability of the H_2_ and O_2_ species mass faction on the electrode–electrolyte interfaces; therefore, the MOLB-type SOFC has lower concentration losses due to the higher availability of the O_2_ and similar H_2_ species concentration.

The H_2_ mass fraction in the anode–electrolyte interface for the MOLB-PPA SOFC and the P-TBFA SOFC is illustrated in Figure 5. The P-TBFA SOFC demonstrates better H_2_ species distribution uniformity compared to the MOLB-PPA SOFC design, where the minimum local species concentration is around 0.09 for the MOLB-PPA SOFC, and 0.126 for the P-TBFA SOFC, and the maximum local species concentration is around 0.417 for the MOLB-PPA SOFC and 0.347 for the P-TBFA SOFC. Moreover, the consumption of H_2_ takes place from the inlet to the outlet of the fuel channel due to the electrochemical reactions required to generate the current density for obtaining the power density desired; therefore, the species concentration of H_2_ is higher at the inlet of the fuel channel, and it is reduced as hydrogen passes through the cell in both geometries. This improvement made by the P-TBFA SOFC is due to the fact that the speed of the fuel increases as the hydrogen passes through the cell due to the trapezoidal baffles that cause a reduction in the cross-section of the cell as the fuel passes. This enhances the replacement of consumed hydrogen in comparison to the MOLB-PPA SOFC.

Figure 6 shows the O_2_ mass fraction distribution in the cathode–electrolyte interface for the MOLB-PPA SOFC and the P-TBFA SOFC. It can be observed, for both geometries, that the oxygen mass fraction decreases from the inlet to the outlet of the air channel of the SOFC. Also, it can be seen on the oxygen mass fraction distributions that for the P-TBFA SOFC, the oxygen mass fraction availability is lower than the MOLB-PPA SOFC. This is due to the higher current density required to obtain the power density desired (9200 A/m^2^ for the P-TBFA SOFC and 7490 A/m^2^ for the MOLB-PPA SOFC). Although the trapezoidal baffles inside the air channel cause a reduction in the cross-section as the air passes, consequently an increase in the velocity of the oxygen inside the air channel, the replacement of the oxygen is less relevant in the P-TBFA SOFC that the enhancement in the replacement of the oxygen consumed due to the embedded porous pipe that is a feed of air in a counter-flow arrangement through the air channel of the MOLB-PPA SOFC. Consequently, this helps obtain higher power density in the MOLB-PPA SOFC than in the P-TBFA SOFC operating at the same current density (Figure 2) due to the higher availability of the oxygen on the cathode–electrolyte interface of the MOLB-PPA SOFC (Figure 6). Finally, the lower values of O_2_ mass fraction distribution of the MOLB-PPA SOFC take place in the lower surface where the inclined plane of the cathode begins (Figure 6a), and in the P-TBFA SOFC, the lowest values of O_2_ mass fraction take place where the anode, electrolyte, cathode, and the collectors are connected (Figure 6b).

Figure 7 shows the steam mass fraction distribution of both geometries. It can be observed, for both geometries, that the steam mass fraction increases from the inlet to the outlet fuel channel of the SOFCs. These behaviors are mainly due to the consumption of H_2_ and O_2_ mass fraction by the electrochemical reaction that takes place throughout the cell (Figure 5 and Figure 6) and due to the current density production (Figure 3). Moreover, the P-TBFA SOFC shows a more homogeneous distribution of H_2_O species than the MOLB-PPA SOFC, which is related to the H_2_ and O_2_ species distributions (Figure 5 and Figure 6). For the MOLB-PPA-type and the P-TBFA-type geometries, the lower values are around 0.581 and 0.652, respectively, and the higher values are around 0.904 and 0.873, respectively.

**Figure 9:**
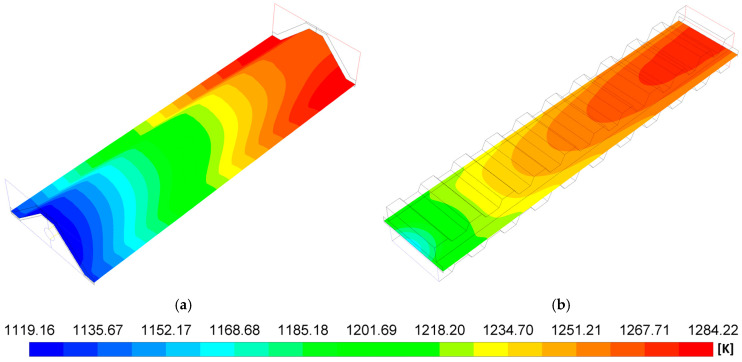
Temperature distributions in the electrode–electrolyte interface: (**a**) MOLB-PPA SOFC and (**b**) P-TBFA SOFC.

The minimum, maximum, and average temperatures in the electrode–electrolyte interface for both geometries are shown in Figure 8. As can be seen, the P-TBFA SOFC and the MOLB-PPA SOFC have a minimum temperature of around 1165 K and 1119 K, respectively. This means an increase of around 43 K in the P-TBFA SOFC. Furthermore, the maximum temperature in the P-TBFA SOFC and the MOLB-PPA SOFC have a maximum temperature of around 1275 K and 1284 K, respectively. This means an increase of around 9 K in the MOLB-PPA SOFC. Thus, the maximum temperature gradient is higher in the MOLB-PPA SOFC, around 165 K, than in the P-TBFA SOFC, around 110 K (Figure 9). As can be noted, the P-TBFA SOFC has a temperature gradient reduction of around 33% in comparison to the MOLB-PPA SOFC to obtain similar power density. This reduction is due mainly to the configurations that have both geometries, and it is possible to observe that the inclusion of the trapezoidal baffles inside fuel and air channels in the planar geometry is more significant than the inclusion of a porous pipe in the air supply channel in a MOLB SOFC to obtain a more homogeneous distribution of temperature, although the average temperature in the electrode–electrolyte interface is higher in the P-TBFA SOFC, around 1245 K than the MOLB-PPA SOFC, around 1216 K. This means an increase around of 29 K in the P-TBFA SOFC. This increase in the average temperature for the P-TBFA SOFC is due to the higher current density necessary, 9200 A/m^2^, to produce the power density desired in comparison to the MOLB-PPA SOFC, 7490 A/m^2^ (Figure 2). An increase in the electrochemical reactions to obtain a higher current density causes an increase in the heat released in the anode–electrolyte interface of the SOFC; consequently, the average temperature of the cell increases.

The temperature distribution in the electrode–electrolyte interface of the MOLB-PPA SOFC and the P-TBFA SOFC is shown in Figure 9. For both geometries, the temperature increases from the inlet to the outlet of the SOFC, as was expected due to the heat released by electrochemical reactions occurring at the anode–electrolyte interface of the SOFC. As can be observed, the P-TBFA SOFC has a temperature gradient reduction in comparison to the MOLB-PPA SOFC, as was discussed in Figure 8. This means that the effect of the baffles inside the planar geometry helps reduce the temperature gradient. The reduction of the cross-section as the height of the baffles increases (Figure 1d) causes higher velocity inside the air channel; consequently, a reduction in the temperature gradient is obtained.

Finally, it can be observed that the inclusion of the trapezoidal baffles inside fuel and air channels in the planar geometry is more significant than the inclusion of a porous pipe in the air supply channel in a MOLB SOFC to obtain a more homogeneous distribution of temperature according to the results shown in Figure 9. On the other hand, according to the results, the inclusion of the porous pipe in the air supply channel in the mono-block-layer-build SOFC causes an increase in the power density due mainly to having a more homogeneous O_2_ mass fraction distribution and more O_2_ availability on the cathode–electrolyte interface (Figure 6).

**Figure 10:**
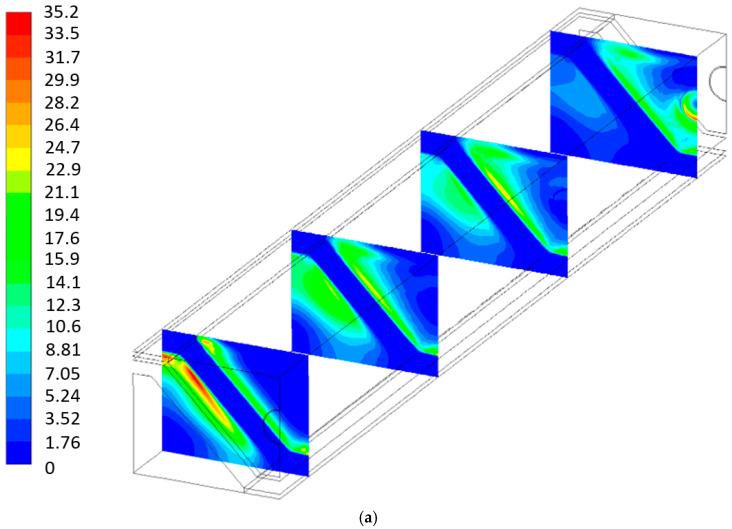

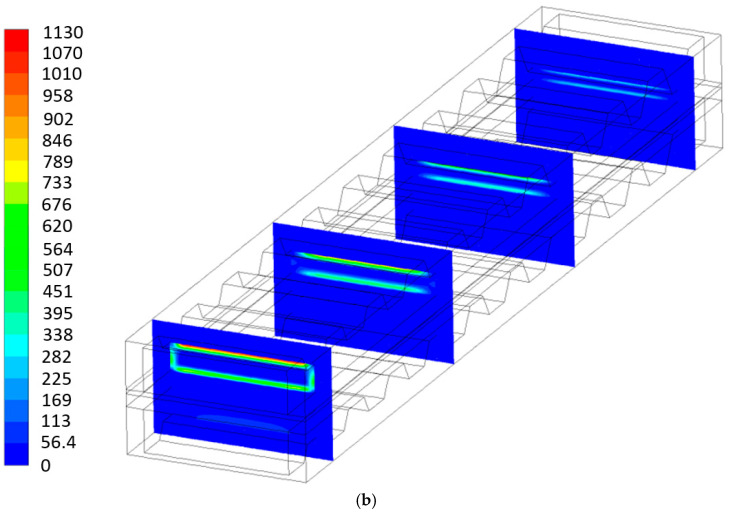
Local entropy generation rate due to heat transfer: (**a**) MOLB-PPA SOFC and (**b**) P-TBFA SOFC [W/K m^3^].

**Figure 11:**
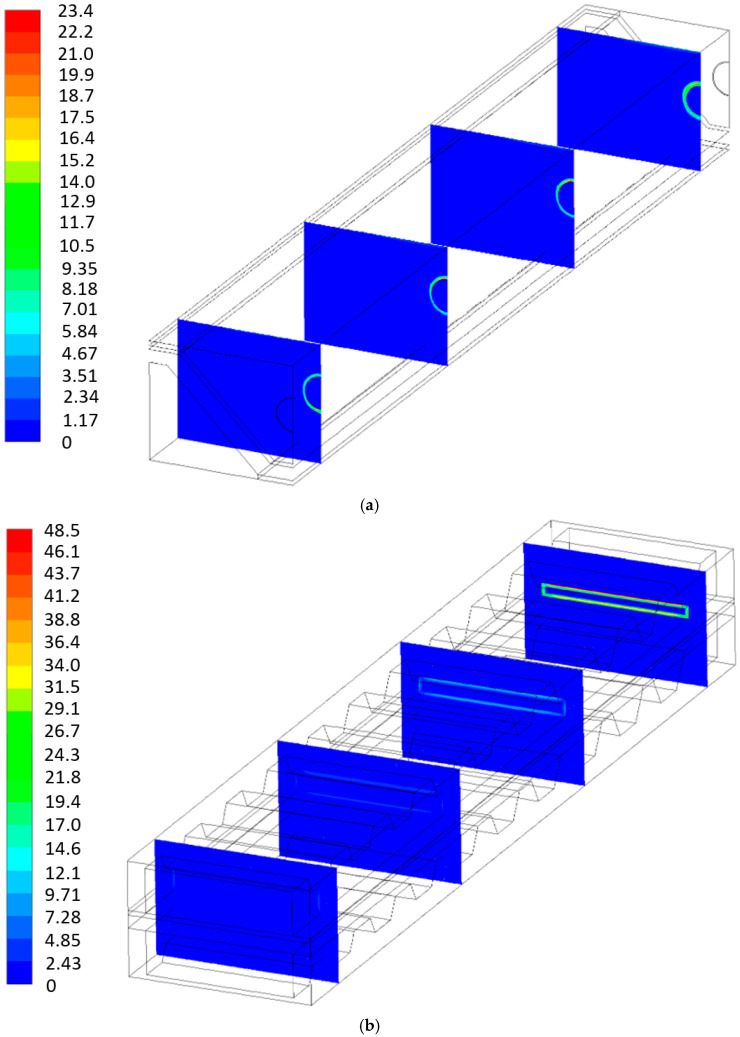
Local entropy generation rate due to fluid flow: (**a**) MOLB-PPA SOFC and (**b**) P-TBFA SOFC [W/K m^3^].

**Figure 12:**
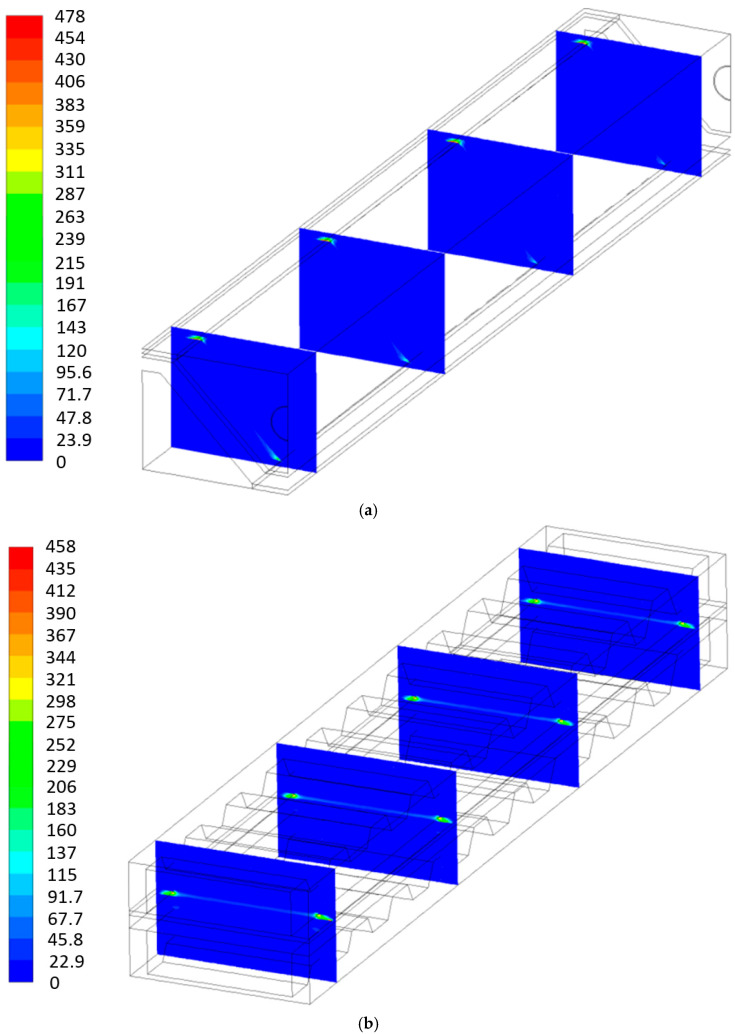
Local entropy generation rate due to mass transfer: (**a**) MOLB-PPA SOFC and (**b**) P-TBFA SOFC [W/K m^3^].

**Figure 13:**
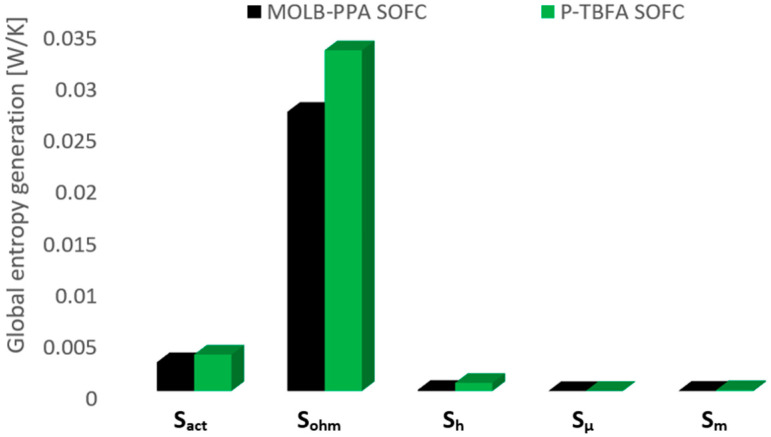
Global entropy generation: MOLB-PPA SOFC and P-TBFA SOFC.

Figure 10, Figure 11, Figure 12 and Figure 13 show the local entropy generation rate due to heat transfer, fluid flow, and mass transfer inside the cell for both geometries in four normal planes throughout the length of the P-TBFA SOFC and MOLB-PPA SOFC at 0.0065, 0.0355, 0.0645 and 0.0935 m. To show properly the local entropy generation rate due to heat transfer and fluid flow phenomena, it was necessary to include its own scale for each geometry; otherwise, the local values of the MOLB-PPA SOFC would be difficult to observe due to the order of magnitude of the values obtained.

Figure 10 shows the entropy generation due to heat transfer for both geometries. According to Equation (23), this phenomenon is mainly related to the temperature gradients within the cell. As can be seen, a lower temperature is observed at the anode–electrolyte interface near the fuel inlet (Figure 9), and since the average inlet temperature of the fuel is lower than this interface temperature, a larger temperature gradient is established between the anode wall and the fuel. As the fuel flows through the channel, it heats up due to chemical and electrochemical reactions occurring on the anode side, reducing the temperature gradient between the fuel and the anode wall. Consequently, the entropy generation due to heat transfer decreases along the channel. Therefore, in the MOLB-type SOFC, a higher entropy generation rate is observed near the fuel inlet, particularly in the cross-sectional plane at 0.0355 m, where the fuel gases are in close contact with the anode surface. As the fuel gases pass along the cell, the entropy generation rate decreases on the fuel side. However, for the air side, the entropy generation rate has lower values at the inlet of the air channel, and the highest values are near the inlet of the porous pipe, a normal plane at 0.0935 m from the inlet air channel. These higher values are attributed to the temperature gradient between the cooler inlet air inside the porous pipe and the higher external temperatures near the cathode–electrolyte interface (Figure 9a). Moreover, for the P-TBFA SOFC, the entropy generation rate due to heat transfer is higher near the inlet of the fuel channel, normal plane at 0.00355 m from the inlet fuel channel, and the highest values are where the fuel gases are near or in contact with the surfaces of the current collectors and the anode. Also, as the fuel passes through the P-TBFA SOFC, the entropy generation rate due to heat transfer decreases.

Figure 11 shows the entropy generation due to fluid flow for both geometries. This phenomenon is related to the viscous effect, the velocity of the air and the fuel, and the geometry. For the MOLB-type SOFC, the higher entropy generation rate can be observed near the wall of the porous pipe throughout the cell, and it is more relevant near the inlet of the air of the porous pipe, a normal plane at 0.0065 m from the inlet of the air of the porous pipe. Meanwhile, for the P-TBFA SOFC, as the cross-section is reduced in the air channel, the entropy generation rate due to the fluid flow increases, and the higher values are near the walls of the current collectors, including the walls of the trapezoidal baffles, and the walls of the cathode. Moreover, for both geometries, the order of magnitude of the entropy generation rate due to fluid flow is less relevant on the anode side, as can be observed. This is mainly due to the velocity considered on the fuel channel as a boundary condition.

The local entropy generation rate distribution due to mass transfer for both geometries can be observed in Figure 12. This entropy generation rate is primarily related to species concentration gradients within the SOFC, as described in Equation (25); therefore, as can be seen, this phenomenon is relevant for the MOLB-PPA SOFC, where anode, electrolyte, cathode, and the current collectors make the corner effect. In these regions, large variations in the concentrations of H_2_, O_2,_ and H_2_O are observed (Figure 5a, Figure 6a, and Figure 7a). For the P-TBFA SOFC, this phenomenon is more relevant on the cathode side, especially where the anode, electrolyte, cathode, and current collectors are connected. Here, larger concentration gradients of O_2_ are observed (Figure 6b). Furthermore, a similar order de magnitude of the values of this phenomenon can be observed for both geometries throughout the cells.

The global entropy generation due to the activation, ohmic, heat transfer, fluid flow, and mass transport losses for both geometries is illustrated in Figure 13. As can be seen, for both geometries, the ohmic losses are predominant. The MOLB-PPA SOFC represents 90.2% of the total of the global entropy generation of the SOFC, and the P-TBFA SOFC represents 88.2% of the total of the global entropy generation of the SOFC. It is worth mentioning that this irreversibility is due to the electrical conductivity of the solid materials, anode, electrolyte, cathode, and current collectors, the temperature of the cell, and the average current density (Equation (27)). In this comparison, the average current density is more relevant. As the average current density is higher, this contribution is higher. In this sense, the entropy generation due to ohmic losses for the P-TBFA SOFC is 22% higher, average current density of 9200 A/m^2^, than the MOLB-PPA SOFC, average current density of 7490 A/m^2^. Moreover, the entropy generation due to mass transport represents 0.12% and 0.25% in the MOLB-PPA SOFC and P-TBFA SOFC, respectively. This irreversibility for the P-TBFA SOFC is 2.64 times higher than that obtained for the MOLB-PPA SOFC. The entropy generation due to fluid flow represents 0.004% and 0.02% in the MOLB-PPA SOFC and P-TBFA SOFC, respectively. For the P-TBFA SOFC, this entropy generation is 6.43 times higher than the obtained for the MOLB-PPA SOFC. Therefore, it can be said that the entropy generation due to mass transport and due to the fluid flow are not significant. Moreover, for the MOLB-PPA SOFC and P-TBFA SOFC, the entropy generation due to the activation losses represents similar percentages of the total global entropy generation of the SOFC, 9.31% for the MOLB-PPA SOFC and 9.43% for the P-TBFA SOFC, and this irreversibility for the P-TBFA SOFC is 26.4% higher than the obtained from the MOLB-PPA SOFC, this irreversibility is due to the material properties and is related to the Buttler–Volmer equation (Equation (13)). Finally, the entropy generation due to heat transfer for the MOLB-PPA SOFC represents 0.41% of the total global entropy generation of the SOFC, and for the P-TBFA SOFC represents 2.12% of the total global entropy generation of the SOFC. However, for the P-TBFA SOFC, the irreversibilities of this phenomenon are 6.43 times higher in comparison to the MOLB-PPA SOFC. Therefore, the total global entropy generation, the sum of the entropy generation due to the activation, ohmic, heat transfer, fluid flow, and mass transfer losses, are 24.75% higher in the P-TBFA SOFC than in the MOLB-PPA SOFC.

## 5. Conclusions

A detailed performance comparison of MOLB-PPA SOFC and P-TBFA SOFC in a SOFC was analyzed. The heat transfer, mass transfer, species transport, and electrochemical reaction were considered in the CFD model. After the analysis, the main conclusions are as follows:

- An increase of around 7.5% in the maximum power density reached for the MOLB-PPA SOFC in comparison to the maximum power density reached for the P-TBFA SOFC was obtained.
- The MOLB-PPA SOFC has an increase in the O_2_ mass fraction on the cathode–electrolyte interface until 24.5% with respect to the P-TBFA SOFC. This helps increase the power density of the MOLB-PPA SOFC.
- The P-TBFA SOFC has a temperature gradient reduction of around 33% in comparison to the MOLB-PPA-type to obtain similar power density. In consequence, the inclusion of the trapezoidal baffles inside fuel and air channels in the planar geometry helps obtain a more homogeneous distribution of temperature in the electrode–electrolyte interface, which could help to avoid failures in the materials of the components due to thermal stresses.
- For current densities lower than 4000 A/m^2^, both geometries have similar performance and the same power density at the same current density.
- For a similar current density of both geometries, higher than 4000 A/m^2^, the inclusion of the porous pipe in the air supply channel in the mono-block-layer-build SOFC causes an increase in the power density due to having a more homogeneous O_2_ mass fraction distribution and higher species concentration of O_2_ in the cathode–electrolyte interface.
- A lower global entropy generation due to heat transfer in the MOLB-PPA SOFC in comparison to the P-TBFA SOFC is obtained.
- The entropy generation due to ohmic losses is predominant for both geometries, and the P-TBFA SOFC is 22% higher than the MOLB-PPA SOFC. The second meaningful contribution of the entropy generation is due to activation losses, and the P-TBFA SOFC is 26.4% higher than the MOLB-PPA SOFC. To reduce these irreversibilities, the average temperature could be increased to reduce the resistivity of the materials, and new materials with improved properties are needed.
- The total irreversibilities are 24.75% higher in the P-TBFA SOFC than in the MOLB-PPA SOFC.
- The global entropy generation, for both geometries, due to fluid flow and mass transport are negligible.

Finally, it can be said that the selection of the geometry of the SOFC has a meaningful impact on the species, temperature, and current density distributions on the electrode–electrolyte interfaces and in the local and the global entropy generation rate due to heat transfer, fluid flow, mass transfer, ohmic losses, and activations losses and in consequence on the performance of the SOFC. Additionally, it is important to note that a simple planar SOFC geometry is generally easier and less expensive to manufacture compared to the geometries analyzed in this study [40,41,42], which could be a key consideration when selecting the most suitable configuration for practical applications.

## Figures and Tables

**Table 1:** Model parameters and material properties [16].

Description	Units	Values
Anode transfer coefficient, $\beta_{a}$	(-)	0.5
Cathode transfer coefficient, $\beta_{c}$	(-)	0.5
Faraday constant, *F*	C mol^−1^	96,487
Anode porosity, $\varepsilon_{a}$	(-)	0.3
Cathode porosity, $\varepsilon_{c}$	(-)	0.3
Anode tortuosity, $\tau_{a}$	(-)	3
Cathode tortuosity, $\tau_{c}$	(-)	3
Anode permeability, $K_{a}$	m^2^	1 × 10^−12^
Cathode permeability, $K_{c}$	m^2^	1 × 10^−12^
Anode thermal conductivity, *k_a_*	W K^−1^ m^−1^	6.23
Cathode thermal conductivity, *k_c_*	W K^−1^ m^−1^	9.6
Electrolyte thermal conductivity, *k_e_*	W K^−1^ m^−1^	2.7
Interconnect thermal conductivity, *k_i_*	W K^−1^ m^−1^	13
Anode specific heat, *Cp_a_*	J kg^−1^ K^−1^	650
Cathode specific heat, *Cp_c_*	J kg^−1^ K^−1^	900
Electrolyte specific heat, *Cp_e_*	J kg^−1^ K^−1^	300
Interconnect specific heat, *Cp_i_*	J kg^−1^ K^−1^	800
Anode density, $\rho_{a}$	kg m^−3^	6200
Cathode density, $\rho_{c}$	kg m^−3^	6000
Electrolyte density, $\rho_{e}$	kg m^−3^	5560
Interconnect density, $\rho_{i}$	kg m^−3^	7700

**Table 2:** Variation in the current density as the number of elements increases.

MOLB-PPA SOFC			P-TBFA SOFC		
Number of Elements	Current Density (A/m^2^)	Variation (%)	Number of Elements	Current Density (A/m^2^)	Variation (%)
37,740	8333.57	--	48,000	8150.67	--
91,200	8551.92	2.62	250,200	8554.92	4.96
150,960	8342.74	2.45	785,650	8093.25	5.39
189,560	8075.46	3.20	1,509,430	8195.74	1.27
290,000	8092.85	0.22	3,000,000	8244.23	0.59
685,320	8097.33	0.06	5,000,000	8240.1	0.05

## Data Availability

The original contributions presented in this study are included in the article. Further inquiries can be directed to the corresponding author(s).

## Nomenclature

*D_ij_*	Mass diffusion coefficient (m^2^ s^−1^)
*D_i,eff_*	Effective mass diffusion coefficient (m^2^ s^−1^)
*h*	Specific enthalpy (J kg^−1^)
j	Current density (A m^−2^)
*j_0_*	Exchange current density (A m^−2^)
${\vec{J}}_{i}$	Diffusive flux of species *j* (kg m^−2^s^−1^)
*k_eff_*	Thermal effective conductivity (W m^−1^ K^−1^)
*M_i_*	Molecular weight of the species *i* (kg mol^−1^)
$n_{e}$	Number of electrons (-)
*P*	Pressure (Pa)
*R*	Universal gas constant (W mol^−1^ K^−1^)
*S_e_*	Energy source term (W m^−3^ s^−1^)
*S_i_*	Species source term (kg m^−3^ s^−1^)
*s_μ_*	Fluid friction entropy generation rate (W K^−1^ m^−3^)
*s_h_*	Heat transfer entropy generation rate (W K^−1^ m^−3^)
*s_m_*	Mass transfer entropy generation rate (W K^−1^ m^−3^)
*s_ohm_*	Chemical reaction entropy generation rate (W K^−1^ m^−3^)
*T*	Temperature (K)
*t*	Time (s)
$\vec{v}$	Velocity (m s^−1^)
*V*	Voltage (V)
$V^{0}$	Standard electric potential (V)
*X_i_*	Molar fraction of species *i* (-)
Greek symbols	
$n_{act}$	Activation potential (V)
$n_{ohm}$	Ohmic loss (V)
$n_{conc}$	Concentration loss (V)
*µ*	Viscosity (m s^−2^)
σ	Electrical conductivity (Ω^−1^ m^−1^)
ϯ	Tortuosity (-)
*ω_i_*	Mass fraction of species *i* (-)
∅	Electrical potential (V)
